# Levels of self-reported depression and anxiety among HIV-positive patients in Albania: a cross-sectional study

**DOI:** 10.3325/cmj.2011.52.622

**Published:** 2011-10

**Authors:** Shane D. Morrison, Vilson H. Banushi, Clea Sarnquist, Valbona H. Gashi, Lars Osterberg, Yvonne Maldonado, Arjan Harxhi

**Affiliations:** 1 Department of Pediatrics, Stanford University School of Medicine, Stanford, CA, USA; 2 Department of Infectious Diseases, University of Tirana School of Medicine, Tirana, Albania; 3 Department of Medicine, Stanford University School of Medicine, Stanford, CA, USA

## Abstract

**Aim:**

To gain an initial perspective of mental health issues facing the Human Immunodeficiency Virus (HIV)-positive population at the University Hospital Center of Tirana (UHCT) HIV/AIDS Ambulatory Clinic.

**Methods:**

From June-August 2009, we conducted semi-structured interviews with 79 patients (93% response rate) at the UHCT HIV/AIDS Ambulatory Clinic. The interviews assessed patient-reported histories of mental health diagnoses, patients’ demographics, and current emotional health status.

**Results:**

The percentage of patients who reported a history of diagnosis of depression or anxiety was high – 62.3% and 82.3%, respectively. Factors associated with a history of depression included having been diagnosed with anxiety (*P* < 0.001), having a higher number of barriers to care (*P* < 0.001), having a higher number of current medical and social needs (*P* < 0.001), or having not obtained antiretroviral therapy (ART) abroad (*P* = 0.004). Factors associated with a history of anxiety included having been on first-line ART (*P* = 0.008), having been diagnosed with HIV for shorter periods of time (*P* = 0.043), having been diagnosed with depression (*P* < 0.001), having a higher number of current medical and social needs (*P* = 0.035), or having not obtained ART abroad (*P* = 0.003).

**Conclusions:**

Mental health problems are widespread among the known HIV-positive patient population in Albania. The high prevalences of anxiety and depression and of dual diagnoses of these conditions suggest the need for more mental health care for HIV-positive patients in Albania.

Mental health is one of the co-morbidities that is often overlooked in treating patients for Acquired Immune Deficiency Syndrome from Human Immunodeficiency Virus (HIV/AIDS) (1-3). In particular, the rates of depression and anxiety are higher than those in the general population (1-6). Depression is second only to substance abuse as the most prevalent psychiatric disorder among HIV-positive patients (5). In the context of HIV/AIDS, depression has also been shown to lead to more social isolation, lower antiretroviral medication adherence, and faster progression to AIDS (7-14). Anxiety, especially among those that have recently been diagnosed with HIV, has been shown to be more prevalent among patients with stress or excess social stigma related to their diagnosis (15-17). Anxiety can also correlate with lower adherence to antiretroviral therapy (ART) and medical recommendations (18,19).

With mental health issues affecting medical treatment of HIV, mechanisms to reduce their burden among HIV-positive patients have been explored. Treatment of depression has been shown to improve adherence to ART along with the quality of life for HIV-positive patients (5,20,21). Community-based group therapy has also been shown to decrease psychiatric symptoms in HIV-positive patients or in regions with high prevalence of HIV, while treatment with ART may reduce both anxiety and depression (22,23). However, with all the advances in the field of mental health, there is still a paucity of data from developing countries (especially Eastern and Central Europe) on the relationship between HIV/AIDS and mental health (18).

With the growing epidemic of HIV in Eastern Europe and possible spread to South Eastern Europe, an understanding of the mental health issues facing HIV-positive patients will be vital for the improvement of medical services and treatment for HIV (18,24-29). This is especially true in countries that have only recently initiated psychological services for HIV positive patients. Albania, which boasts a low prevalence of HIV, is one such country that initiated psychological services soon after the introduction of ART in 2004 (30,31). High levels of risky behavioral patterns (including low condom usage and high rates of needle sharing among injection drug users), the recent sociopolitical changes, and the under-resourced prevention and surveillance capabilities, have placed the Albanian population at risk for a rising local HIV epidemic (30-34). In fact, previous studies have suggested that the prevalence of HIV in Albania may be 150-fold the current Ministry of Health estimate (35,36). Thus, an initial patient-driven assessment of the mental health issues of patients under HIV/AIDS medical care in Albania is warranted. In this study, we examined the prevalence of HIV-positive patients’ self-reported histories of mental health diagnoses in Albania. This study also examined effects of ART on mental health and associations with depression and anxiety.

## Methods

### Setting

The setting for this study was the University Hospital Center of Tirana (UHCT) HIV/AIDS Ambulatory Clinic. This clinic is the only site in Albania offering ART, and it initiated its program in 2004 (30,31). ART is offered free of charge. All known patients in need of HIV/AIDS medical care in Albania attain their care at this facility. Aspects of the clinic were described previously (37).

### Participants

During the course of this study (June-August 2009), there were 85 patients in Albania who sought care at the UHCT HIV/AIDS Ambulatory Clinic. With only 359 patients having ever been diagnosed in Albania and approximately 100 patients actively under care at UHCT HIV/AIDS Ambulatory Clinic, this represented the majority of patients under HIV/AIDS medical care in Albania during the study period. Of the patients who sought care, 79 (93%) agreed to participate in the study.

### Instrument

The instrument (web-extra material)(web extra material 1)  was developed in conjunction with Stanford University School of Medicine, the UHCT HIV/AIDS Ambulatory Clinic, and the Ministry of Health in Albania. Parts of the instrument and its implementation were described previously (33,37-39). Both the Stanford University Institutional Review Board and the Albanian Ethical Committee approved the study.

Patients were asked to self-report histories of mental health diagnoses following their HIV diagnosis. Medical record review to confirm reported diagnoses was not undertaken. Since this survey was given in the form of semi-structured interviews (due to low literacy levels of patients), patients were given the options of depression, anxiety, dementia, bipolar disorder, or other. A psychologist directed the semi-structured interviews, so elaboration was possible when needed. Patients were also asked about their current emotional health and whether initiating treatment with ART had altered it. Again the answer choices were presented in a multiple-choice format.

### Data analysis

Data were electronically stored and 10% of the data was checked for errors with none found. Patient-reported histories of mental health diagnoses were presented as absolute number and percentages. Current emotional health and changes in emotional health since initiating ART were presented in the same manner. Demographics of the patients were also recorded and presented previously (37). The association of categorical variables with depression or anxiety was evaluated with χ^2^ tests unless any cell in the contingency tables had n <5, in which case Fischer exact tests were used. For continuous variables, comparisons were done with the *t* test or Wilcoxon rank sums test depending on the normality of distribution of the data. Indices for barriers to care and current medical and social needs were developed. For each of these indices, the total number of barriers to care (n = 0-18) or current medical and social needs (n = 0-9) was added for each individual patient. The questions that were used to develop the indices can be seen in section 1 of the web-extra material; any “yes” answer was treated as a +1 response and other answers were treated as 0 in the respective index. The components of each of the indices were reported previously (37). These indices were then treated as continuous variables in further analysis.

## Results

The majority of patients reported that their current emotional health was excellent, good, or fair, with only 11.4% stating that it was poor. The majority of patients (78.5%) also reported that their emotional health had improved since initiating ART – 18.2% stated their emotional health was the same and 2.6% stated that it was a little worse (Table 1). The prevalence of both depression (62.3%) and anxiety (82.3%) was high, while that of dementia (12.8%) and bipolar disorder (0.0%) was low or absent (Table 1).

**Table 1:** Mental health characteristics of patients (n = 79) receiving medical services at University Hospital Center of Tirana HIV/AIDS Ambulatory Clinic. Patients were allowed to abstain from answering any of the questions in the survey, thus some of the answers have less than 79 respondents

Characteristic	No. (%)
**Current mental health:**	
excellent	1 (1.3)
good	23 (29.1)
fair	46 (58.2)
poor	9 (11.4)
Change in mental health since starting treatment:*	
much better	16 (20.1)
a little better	45 (58.4)
about the same	14 (18.2)
a little worse	2 (2.6)
much worse	0 (0.0)
**Self-reported mental illness since HIV/AIDS diagnosis:**	
anxiety	65 (82.3)
bipolar disorder^†^	0 (0.0)
dementia^‡^	10 (12.8)
depression*	48 (62.3)

*Only 77 respondents.

†Only 76 respondents.

‡Only 78 respondents.

Bivariate analysis showed that patients with a history of depression following HIV diagnosis were more likely to have an anxiety diagnosis (*P* < 0.001), have a higher number of barriers to care (*P* < 0.001), have a higher number of current medical and social needs (*P* < 0.001), and/or have not obtained their ART abroad (*P* = 0.004) (Table 2). Similar analysis showed that those with diagnoses of anxiety following HIV diagnosis were more likely to have been on first-line ART (*P* = 0.008), have been diagnosed with HIV for shorter period of time (*P* = 0.03), have a history of depression (*P* < 0.001), have a higher number of current medical and social needs (*P* = 0.05), and/or have not obtained their ART abroad (*P* = 0.003) (Table 3).

**Table 2:** Associations with depression of patients receiving medical services at the University Hospital Center of Tirana HIV/AIDS Ambulatory Clinic. Patients were allowed to abstain from answering any question, thus not all responded to every question

	No. (%) of patients or mean ± standard deviation		
Characteristic	with depression (n = 48)	without depression (n = 29)	*P*
Age, years	38.4**±**11.1	40.5**±**12.1	0.641
Female	19 (67.7)	9 (32.1)	0.450
Heterosexual	45 (93.7)	27 (93.1)	1.000
Married	27 (56.3)	14 (48.3)	0.497
Completed secondary school or higher (8 classes)	31 (64.6)	17 (58.6)	0.601
Muslim	37 (77.1)	24 (82.8)	0.773
Number of years abroad	2.2 ± 4.3	3.5 ± 5.3	0.524
Obtained antiretroviral therapy abroad*	0 (0.0)	5 (21.7)	0.004
First-line therapy*	27 (62.8)	9 (39.1)	0.066
Employed/student/retired	21 (43.7)	15 (51.7)	0.497
Number of years since HIV diagnosis	3.9 ± 2.8	5.3 ± 3.2	0.546
Anxiety	48 (100)	15 (51.7)	<0.001
Dementia	7 (14.6)	2 (6.9)	0.470
Household members with HIV^†^	13 (27.7)	6 (20.7)	0.496
Barrier Index^‡^	8.6 ± 2.5	5.6 ± 3.3)	<0.001
Need Index^§^	7.2 ± 2.0	4.1 ± 2.5	<0.001

*43 patients with depression and 23 patients without depression.

†47 patients with depression and 29 patients without depression.

‡Barrier Index is defined in the methods section. It includes barriers experienced by patients in seeking HIV medical care..

§Need Index is defined in the methods section. It includes current needs that patients are having with regard to medical and daily life..

**Table 3:** Associations with anxiety of patients receiving medical services at University Hospital Center of Tirana HIV/AIDS Ambulatory Clinic

	No. (%) of patients or mean ± standard deviation		*P*
Characteristic	with anxiety (n = 65)	without anxiety (n = 14)	
Age, years	38.6 ± 10.9	43.2 ± 13.4	0.338
Female	24 (36.9)	4 (28.6)	0.760
Heterosexual	61 (93.9)	13 (92.9)	1.000
Married	37 (56.9)	6 (42.9)	0.338
Completed secondary school or higher (8 classes)	43 (66.2)	7 (50.0)	0.255
Muslim	53 (81.5)	10 (71.4)	0.466
Number of years abroad	2.3 ± 4.1	4.4 ± 6.4	0.604
Obtained antiretroviral abroad*	1 (1.8)	4 (33.3)	0.003
First-line therapy*	35 (62.5)	2 (16.7)	0.008
Employed/student/retired	29 (44.6)	8 (57.1)	0.394
Number of years since HIV diagnosis	4.2 ± 2.6	5.5 ± 4.4	0.043
Depression^†^	48 (76.2)	0 (0.0)	<0.001
Dementia^‡^	9 (14.1)	1 (7.1)	0.680
Household members with HIV^‡^	18 (28.1)	2 (14.3)	0.500
Barrier index^§^	7.7 ± 3.0	6.1 ± 3.7	0.135
Need index^II^	6.3 ± 2.6	4.6 ± 2.7	0.035

*56 patients with anxiety and 12 patients without anxiety.

†63 patients with anxiety and 14 patients without anxiety.

‡64 patients with anxiety and 14 patients without anxiety.

§Barrier Index is defined in the methods section. It includes barriers experienced by patients in seeking HIV medical care.

IINeed Index is defined in the methods section. It includes current needs that patients are having with regard to medical and daily life.

## Discussion

The reported level of psychiatric morbidity among HIV-positive patients in Albania is high relative to that among the general population – 26.2% (40). Moreover, depression levels among HIV-positive patients of the magnitude reported in this study have been noted in only a small number of earlier studies in developing countries. Anxiety levels are also on the high end of what has been seen previously (18,41). Such high levels of patient-reported diagnosis of mental health issues offers cause for concern within this population, as previous studies have shown that they can affect patient outcomes (5,7-14,20,21). However, HIV-positive patients in Albania generally present at later stages of infection with other AIDS-related co-morbidities, which may influence the levels of depression and anxiety (Harxhi A, unpublished data). Psychological services for HIV-positive patients, which have only recently been initiated in Albania, are underutilized and patients generally do not feel psychological care is necessary (30,31,37). Even though outcomes, as associated with mental health, were not assessed in this study, scaling up of the psychological and mental health services in Albania may be beneficial. Indeed, previous studies in developing countries have shown that proper interventions, including a cognitive-behavioral group plan and community-driven group interpersonal psychotherapy, can reduce depressive symptoms and may lead to a better quality of life for patients with HIV or patients in regions with a high prevalence of HIV (23,42).

A number of factors were associated with patient-reported diagnoses of depression and/or anxiety. Both histories of depression and anxiety frequently coexisted in patients; in fact, all patients who reported having a history of depression diagnosis also had a history of anxiety diagnosis. This finding suggests that anxiety or depression following HIV diagnosis may be co-morbid conditions. Thus, early mental health intervention may prevent or ameliorate the symptoms of both depression and anxiety (43). The more medical and social needs that patients had, the more likely they were to have histories of diagnoses of either depression or anxiety. We have shown previously that HIV-positive patients in Albania have high levels of current medical and social needs, so interventions at this level may minimize the cases of depression and anxiety among this patient population (37). Indeed, many countries in Eastern and Central Europe are developing mechanisms to approach the high burden of patients’ medical and social needs (44). Another interesting finding was the effect that having obtained ART abroad had on patient-reported histories of diagnoses of depression and anxiety. Patients who had not obtained ART abroad were more likely to have a history of diagnosis of anxiety and/or depression. These observations may be related to patients’ lack of faith in the medical treatments in Albania or greater availability of mental health services outside of Albania (45,46). However, more in-depth exploration on the impact of obtaining ART abroad on the mental health of patients with HIV/AIDS is necessitated.

A history of diagnosis of depression was also associated with increased numbers of barriers to care that were experienced by HIV-positive patients when seeking medical attention. Previous research has noted that barriers to care in Eastern and Central Europe are numerous, and navigating these obstacles can have a variety of adverse effects (37,47-49). Minimizing these barriers, especially the social stigma related to HIV/AIDS, in Albania could function to reduce the prevalence of depression among this population. A history of diagnosis of anxiety was also associated with being on first-line therapy and having been diagnosed with HIV for shorter periods of time. With the high levels of social stigma related to HIV/AIDS in Albania, patients with more recent diagnoses may be dealing with the stress of their diagnosis that could be leading to anxiety (15). As mentioned above, minimization of social stigma may reduce anxiety in patients. Still, more in-depth studies are required to determine the causality of these associations and the proper interventions to implement.

The majority of patients reported that their emotional health was fair, possibly indicating the necessity for more collaboration with psychologists or psychiatrists during the delivery of medical care to HIV-positive individuals in Albania. Patients also reported that treatment with ART generally improved their emotional health. Treatment with ART can improve the physical ailments of AIDS-related medical problems in patients, which may be leading to their increased emotional health status (5,18). In addition, since there is pre-counseling with an on-site psychologist prior to initiating ART, this may be more evidence indicating that interaction with a mental health professional can lead to improved emotional health in Albania.

There are a number of limitations that exist in this study. The largest limitation is that histories of mental health diagnoses were self-reported. However, since only a limited number of data are currently available, this study provides a nonetheless important initial perspective on the mental health status of HIV-positive patients in Albania. Moreover, patient-reported instruments tend to produce an under-reporting of the prevalence of mental health diagnoses, yet reported values are still high (50). It may also be the case that over-reporting of the prevalence of mental health diagnoses occurred, if, for example, symptoms were equated with diagnoses. This scenario tends to occur in situations when patients and/or providers have low mental health literacy, which could be the case in Albania (51). Studies with validated instruments to assess mental health have been successful in developing countries, so this is the next step that needs to be pursued in this population (18). Further structured clinical interviews with professionally trained psychiatrists are needed to validate the findings of this study. Participants in the study were not queried on whether their mental health diagnoses were recurrent or new. Being able to further parse the population into those suffering from recurrent or new mental health diagnoses may allow differing clinical approaches to managing these patients and will need to be addressed in future studies. The small sample size of this study population (especially of those on ART) and the fact that these patients are actively seeking and receiving HIV medical care also limits the generalizability of the results. However, this study covered the majority of patients under HIV-related medical care in Albania, so it gives an initial insight into the mental health status of the current population and an estimate of what could occur in the future.

With the HIV epidemic continuing to evolve in Eastern and Central Europe, countries in this region need to develop innovative and flexible interventions to complement their local epidemics. Assessing and treating mental health issues has become an important component of HIV/AIDS policy and has been shown to improve patient quality of life as well as outcomes (5,18,44). The number of HIV-positive patients with self-reported histories of mental health diagnoses in Albania is high, so addressing these issues for patients in the future may be vital in combating a local epidemic. Early recognition of these diseases and proper intervention could aid in maintaining the low HIV prevalence in Albania (52,53).

## References

[1] Lyketsos CG, Federman EB (1995). Psychiatric disorders and HIV infection: impact on one another.. Epidemiol Rev.

[2] Owe-Larsson B, Sall L, Salamon E, Allgulander C (2009). HIV infection and psychiatric illness.. Afr J Psychiatry (Johannesbg).

[3] Stober DR, Schwartz JA, McDaniel JS, Abrams RF (1997). Depression and HIV disease: prevalence, correlates, and treatment.. Psychiatr Ann.

[4] Prince M, Patel V, Saxena S, Maj M, Maselko J, Phillips MR (2007). No health without mental health.. Lancet.

[5] Rabkin JG (2008). HIV and depression: 2008 review and update.. Curr HIV/AIDS Rep.

[6] Remien RH, Mellins CA (2007). Long-term psychosocial challenges for people living with HIV: let's not forget the individual in our global response to the pandemic.. AIDS.

[7] Mayne TJ, Vittinghoff E, Chesney MA, Barrett DC, Coates TJ (1996). Depressive affect and survival among gay and bisexual men infected with HIV.. Arch Intern Med.

[8] Atkinson JH, Heaton RK, Patterson TL, Wolfson T, Deutsch R, Brown SJ (2008). Two-year prospective study of major depressive disorder in HIV-infected men.. J Affect Disord.

[9] Page-Shafer K, Delorenze GN, Satariano WA, Winkelstein W (1996). Comorbidity and survival in HIV-infected men in the San Francisco Men's Health Survey.. Ann Epidemiol.

[10] Evans DL, Leserman J, Perkins DO, Stern RA, Murphy C, Zheng B (1997). Severe life stress as a predictor of early disease progression in HIV infection.. Am J Psychiatry.

[11] Bouhnik AD, Préau M, Vincent E, Carrieri MP, Gallais H, Lepeu G (2005). Depression and clinical progression in HIV-infected drug users treated with highly active antiretroviral therapy.. Antivir Ther.

[12] Ickovics JR, Hamburger ME, Vlahov D, Schoenbaum EE, Schuman P, Boland RJ (2001). Mortality, CD4 cell count decline, and depressive symptoms among HIV-seropositive women: longitudinal analysis from the HIV Epidemiology Research Study.. JAMA.

[13] Green G, Smith R (2004). The psychosocial and health care needs of HIV-positive people in the United Kingdom: a review.. HIV Med.

[14] Williams P, Narciso L, Browne G, Roberts J, Weir R, Gafni A (2005). The prevalence, correlates, and costs of depression in people living with HIV/AIDS in Ontario: implications for service directions.. AIDS Educ Prev.

[15] Au A, Chan I, Li P, Chung R, Po LM, Yu P (2004). Stress and health-related quality of life among HIV-infected persons in Hong Kong.. AIDS Behav.

[16] Chandra PS, Deepthivarma S, Jairam KR, Thomas T (2003). Relationship of psychological morbidity and quality of life to illness-related disclosure among HIV-infected persons.. J Psychosom Res.

[17] Eller LS, Mahat G (2003). Psychological factors in Nepali former commercial sex workers with HIV.. J Nurs Scholarsh.

[18] Collins PY, Holman AR, Freeman MC, Patel V (2006). What is the relevance of mental health to HIV/AIDS care and treatment programs in developing countries? A systematic review.. AIDS.

[19] Stout BD, Leon MP, Niccolai LM (2004). Nonadherence to antiretroviral therapy in HIV-positive patients in Costa Rica.. AIDS Patient Care STDS.

[20] Ferrando SJ, Freyberg Z (2008). Treatment of depression in HIV positive individuals: a critical review.. Int Rev Psychiatry.

[21] Repetto MJ, Petitto JM (2008). Psychopharmacology in HIV-infected patients.. Psychosom Med.

[22] Jelsma J, Maclean E, Hughes J, Tinise X, Darder M (2005). An investigation into the health-related quality of life of individuals living with HIV who are receiving HAART.. AIDS Care.

[23] Bolton P, Bass J, Neugebauer R, Verdeli H, Clougherty KF, Wickramaratne P (2003). Group interpersonal psychotherapy for depression in rural Uganda: a randomized controlled trial.. JAMA.

[24] UNAIDS/World Health Organization. 2007 AIDS Epidemic Update. Geneva, (Switzerland): UNAIDS/World Health Organization; 2007.

[25] UNAIDS/World Health Organization. Fact Sheet on HIV/AIDS for Eastern Europe and Central Asia. Geneva (Switzerland): UNAIDS/World Health Organization; 2007.

[26] World Health Organization. Epidemiological fact sheets on HIV and AIDS, a 2008 update. Copenhagen: World Health Organization; 2008.

[27] UNAIDS/World Health Organization. Fact Sheet on Global HIV/AIDS. Geneva (Switzerland): UNAIDS/World Health Organization; 2009.

[28] UNAIDS. United Nations AIDS Epidemic Update. Geneva (Switzerland): UNAIDS; 1999.

[29] World Health Organization. World Health Statistics 2008. Geneva (Switzerland): World Health Organization; 2008.

[30] Ministry of Health (Republic of Albania). Albania Country Progress Report. Tirana: Ministry of Health (Republic of Albania); 2007.

[31] Ministry of Health (Republic of Albania). Let's keep Albania a low HIV prevalence country: the national strategy of prevention and control of HIV/AIDS in Albania 2004 - 2010. Tirana: Ministry of Health (Republic of Albania); 2003.

[32] UNICEF. Rapid assessment and response - Tirana. Tirana: UNICEF; 2004.

[33] USAID, Ministry of Health. (Republic of Albania), and Institute of Public Health. Albania. Behavioral and biological surveillance study report. Tirana: USAID, Ministry of Health (Republic of Albania), and Institute of Public Health. Albania; 2006.

[34] UNICEF. Rapid assessment and response on HIV/AIDS among especially vulnerable young people in Albania: a country report. Tirana: UNICEF; 2002.

[35] Ciccozzi M, Gori C, Boros S, Ruiz-Alvarez MJ, Harxhi A, Dervishi M (2005). Molecular diversity of HIV in Albania.. J Infect Dis.

[36] Salemi M, de Oliveira T, Ciccozzi M, Rezza G, Goodenow MM (2008). High-resolution molecular epidemiology and evolutionary history of HIV-1 subtypes in Albania.. PLoS ONE.

[37] Morrison SD, Banushi VH, Sarnquist C, Gashi VH, Osterberg L, Maldonado Y (2011). Barriers to care and current medical and social needs of HIV-positive patients in Albania.. Cent Eur J Public Health.

[38] Lee BW, Sathyan P, John RK, Singh K, Robin AL (2008). Predictors of and barriers associated with poor follow-up in patients with glaucoma in South India.. Arch Ophthalmol.

[39] Simoni JM, Kurth AE, Pearson CR, Pantalone DW, Merrill JO, Frick PA (2006). Self-report measures of antiretroviral therapy adherence: A review with recommendations for HIV research and clinical management.. AIDS Behav.

[40] Bilanakis N, Kaci M, Malamas M (2001). Psychiatric morbidity in an urban area of Albania. A community survey.. Eur J Psychiatry.

[41] Mfusi S, Mahabeer M. (2000). Psychosocial adjustment of pregnant women infected with HIV/AIDS in South Africa. Journal of Psychology in Africa (south of the Sahara, the Caribbean, and Afro-Latin America).

[42] Chan I, Kong P, Leung P, Au A, Li P, Chung R (2005). Cognitive-behavioral group program for Chinese heterosexual HIV-infected men in Hong Kong.. Patient Educ Couns.

[43] Rapaport MH (2001). Prevalence, recognition, and treatment of comorbid depression and anxiety.. J Clin Psychiatry.

[44] Lazarus JV, Laukamm-Josten U, Atun RA, Liljestrand J, Vase I, Matic S (2008). Encouraging innovation: ten research priorities for achieving universal access to HIV/AIDS prevention, treatment and care in Europe by 2010.. Cent Eur J Public Health.

[45] Manfredi R, Calza L, Chiodo F (2003). HIV-infected immigrants from non-European Union countries and antiretroviral treatment: comparison of epidemiologic, clinical, and therapeutic variables according to patient sex.. J Acquir Immune Defic Syndr.

[46] Saracino A, El-Hamad I, Prato R, Cibelli DC, Tartaglia A, Palumbo E (2005). Access to HAART in HIV-infected immigrants: a retrospective multicenter Italian study.. AIDS Patient Care STDS.

[47] Amirkhanian YA, Kelly JA, McAuliffe TL (2003). Psychosocial needs, mental health, and HIV transmission risk behavior among people living with HIV/AIDS in St Petersburg, Russia.. AIDS.

[48] Burruano L, Kruglov Y (2009). HIV/AIDS epidemic in Eastern Europe: recent developments in the Russian Federation and Ukraine among women.. Gend Med.

[49] Mimiaga MJ, Safren SA, Dvoryak S, Reisner SL, Needle R, Woody G (2010). We fear the police, and the police fear us”: structural and individual barriers and facilitators to HIV medication adherence among injection drug users in Kiev, Ukraine.. AIDS Care.

[50] Dodd S, Williams LJ, Jacka FN, Pasco JA, Bjerkeset O, Berk M (2009). Reliability of the Mood Disorder Questionnaire: comparison with the Structured Clinical Interview for the DSM-IV-TR in a population sample.. Aust N Z J Psychiatry.

[51] Lewis G, Pelosi AJ, Araya R, Dunn G (1992). Measuring psychiatric disorder in the community: a standardized assessment for use by lay interviewers.. Psychol Med.

[52] Pence BW (2009). The impact of mental health and traumatic life experiences on antiretroviral treatment outcomes for people living with HIV/AIDS.. J Antimicrob Chemother.

[53] Psaros C, Geller PA, Aaron E (2009). The importance of identifying and treating depression in HIV infected, pregnant women: a review.. J Psychosom Obstet Gynaecol.

