# Supplementary material for: Levels of self-reported depression and anxiety among HIV-positive patients in Albania: a cross-sectional study
Source: Croat Med J. 2011 Oct;52(5):622–7. doi: 10.3325/cmj.2011.52.622 (PMC3195971; doi:10.3325/cmj.2011.52.622)
Supplement: Supplementary material [file CroatMedJ_52_s002.pdf]

This survey is intended to improve the HIV/AIDS services that are offered by the Ministry of Health and Tirana Hospital. **All questions answered in the following survey will remain anonymous and will NOT have any impact on your current or future visits.** We need to know the experiences of our patients and your honesty is invaluable in this process. We appreciate your time and input as we try to improve our services.

Directions: Please check the appropriate boxes. If you answer "other" to any of the following questions **please provide details. Be as specific as possible.** If you cannot remember or feel uncomfortable with any question please draw a line through the question.

CONTINUE ON TO THE NEXT PAGE

Section I – Barriers to Care

1. Currently or in the past 12 months, have you needed any of these services related to your HIV/AIDS infection?:

	Have you needed this service in the past 12 months?	If yes, Have you been able to get this service in the past 12 months?
HIV medical services	☐ Yes → ☐ No ☐ Don't know what this means	☐ Yes ☐ No
Psychologist or psychiatric services	☐ Yes → ☐ No ☐ Don't know what this means	☐ Yes ☐ No
Assistance in finding a doctor for ongoing medical services	☐ Yes → ☐ No ☐ Don't know what this means	☐ Yes ☐ No
Assistance in finding shelter or housing	☐ Yes → ☐ No ☐ Don't know what this means	☐ Yes ☐ No
Assistance with finding meals or food	☐ Yes → ☐ No ☐ Don't know what this means	☐ Yes ☐ No
Assistance with completing daily activities or chores	☐ Yes → ☐ No ☐ Don't know what this means	☐ Yes ☐ No
Transportation assistance	☐ Yes → ☐ No ☐ Don't know what this means	☐ Yes ☐ No
Childcare assistance	☐ Yes → ☐ No ☐ Don't know what this means	☐ Yes ☐ No
Education or information on HIV risk prevention	☐ Yes → ☐ No ☐ Don't know what this means	☐ Yes ☐ No

CONTINUE ON TO THE NEXT PAGE

2. Below is a list of problems that you may have had when trying to **get or use** HIV/AIDS medical services. If you experienced any of the following problems, mark "Yes." If you did not have any of the problems did not affect you, mark "No." If you do not know if the problem affected you, mark "Don't Know."

	Yes	No	Don't Know
Did not know that I could get medical help for HIV/AIDS	☐	☐	☐
Did not know where I could find medical help for HIV/AIDS	☐	☐	☐
Too sick to get medical help	☐	☐	☐
Did not think that HIV/AIDS required medical help	☐	☐	☐
Mental problems (like depression, anxiety, dementia, etc.) kept me from getting medical help for HIV/AIDS	☐	☐	☐
People at the hospital or clinic made me feel worse about myself for having HIV/AIDS	☐	☐	☐
People at the hospital or clinic treated me unfairly because of my HIV/AIDS	☐	☐	☐
My doctor or nurse did not know about HIV/AIDS	☐	☐	☐
I had to wait too long to see a doctor for HIV/AIDS	☐	☐	☐
I was afraid to get in trouble with the law and/or police	☐	☐	☐
Did not want people to know I had HIV/AIDS	☐	☐	☐
I was afraid I would lose my job and/or house because of HIV/AIDS			
No transportation available	☐	☐	☐
No childcare available	☐	☐	☐
I was not eligible for the HIV/AIDS medical help	☐	☐	☐
There was too much paperwork	☐	☐	☐
The services cost too much money	☐	☐	☐
There are too many instructions I have to follow	☐	☐	☐
Other (specify)_________________________	☐	☐	☐

3. Which of the above was the most difficult barrier for you to get through during your search for HIV/AIDS related care?:____

CONTINUE ON TO THE NEXT PAGE

The next section of the questionnaire asks about the HIV-related medications that you have taken.

Most people with HIV have many pills to take at different times during the day.
Many people find it hard to always remember to take their pills:

- Some people get busy and forget to carry their pills with them.
- Some people find it hard to take their pills according to all the instructions, such as "with meals," "on an empty stomach," "every 8 hours," or "with plenty of fluids."
- Some people decide to skip pills to avoid side effects or just not be taking pills that day.

We need to understand how people with HIV are really doing with their medications. Please tell us what you are **actually** doing. Don't worry about telling us you don't take all of your pills. We need to know what is really happening, not what you think we "want to hear." Remember everything you tell us is anonymous and will not affect your visits in any way.

Current Medications		
Medication Name	Number of pills each time (pills each dose)	Number of times per day (doses per day)

CONTINUE ON TO THE NEXT PAGE

Section II - Adherence

1. Are you currently on antiretroviral therapy?
 - Yes
 - No (**Go to section 3**)

 a) If yes, what month and year did you start your therapy?

Month Year

 b) Have you **ALWAYS** received your medication for free from the Ministry of Health?
 - Yes (**Go to question 2**)
 - No

 c) If no, where did you get your medication? **(Select all that apply)**
 - Paid with own money in Albania
 - Paid with own money abroad
 - Medical insurance paid for medication in Albania
 - Medical insurance paid for medication abroad
 - Free medication program in Albania (Not Ministry of Health)
 - Free program abroad
 - Other:__________________

2. Taking all of your HIV medication is difficult. Below asks about the medications that you may have **MISSED** taking over the last four days. If you did not miss any doses, write a zero (0) in the box. Note that the table asks about DOSES not PILLS. **If you took only a portion of a dose on one or more of these days, please report the dose(s) as being missed. See page 4 for current medication doses.**

HOW MANY DOSES DID YOU **MISS**:				
MEDICATION NAME	Yesterday	2 days ago	3 days ago	4 days ago

3. Most medications need to be taken on a schedule, such as “2 times a day” or “3 times a day” or “every 8 hours.” How closely did you follow your specific schedule over the last four days?
 - Never
 - Some of the time
 - About half of the time
 - Most of the time
 - All of the time

CONTINUE ON TO THE NEXT PAGE

4. Do any of your medications have special instructions, such as “take with food” or “on an empty stomach” or “with plenty of fluids”?

☐ Yes (**Go to question 4B**)
☐ No (**Go to question 5**)

B) If yes, how often did you follow those special instructions over the last **four** days?

☐ Never
☐ Some of the time
☐ About half of the time
☐ Most of the time
☐ All of the time

5. When was the last time you missed **ANY** of your medications?

☐ Within past 2 days
☐ Within past 2 weeks
☐ 2-4 weeks ago
☐ 1-3 months ago
☐ More than 3 months ago
☐ Never skip medications

6. During the past four days, on how many days have you missed taking **ALL** of your doses?

☐ None
☐ One day
☐ Two days
☐ Three days
☐ Four days

7. Put a mark on the line below at the point that shows your best guess about how much of your prescribed HIV medication you have taken in the last month. Most people do not take 100% of their medication.

Examples: 0% means you have not taken any of your medication
50% means you have taken half of your medication
100% means you have taken every single dose of your medication

0 10 20 30 40 50 60 70 80 90 100

CONTINUE ON TO THE NEXT PAGE

8. People may miss taking their medications for various reasons. Here is a list of possible reasons why you may miss taking your medications. Have you **ever missed taking your medications** because you: (**Please check one box for each question**)

	**Yes**	**No**	**Don't Know**
Were away from home?	☐	☐	☐
Were busy with other things?	☐	☐	☐
Simply Forgot?	☐	☐	☐
Had too many pills to take?	☐	☐	☐
Wanted to avoid side effects?	☐	☐	☐
Did not want others to notice you taking medication?	☐	☐	☐
Had a change in daily routine?	☐	☐	☐
Felt like the drug was harmful/toxic?	☐	☐	☐
Fell asleep/slept through dose time?	☐	☐	☐
Felt sick or ill?	☐	☐	☐
Felt depressed/overwhelmed?	☐	☐	☐
Had problem taking pills at specified times (with meals, on empty stomach, etc)?	☐	☐	☐
Ran out of pills?	☐	☐	☐
Felt better?	☐	☐	☐
Other:_______________________________________	☐	☐	☐

9. Which of the above was the most **IMPORTANT** reason in for missing your medication?:______________________________________

10. Which of the following would best help you to not miss any doses of your medication:

	**Yes**	**No**	**Don't Know**
Having a reminder of when to take medication	☐	☐	☐
More education on the reasons for taking medication	☐	☐	☐
More education on the seriousness of HIV/AIDS	☐	☐	☐
Not so many pills to take	☐	☐	☐
Could get medication for free near home	☐	☐	☐
Other:____	☐	☐	☐

11. Which of the above would be the most **IMPORTANT** in helping you not to miss any doses of your medication?:____

CONTINUE ON TO THE NEXT PAGE

Section III – Demographics

1. What is your sex/gender?
   - Male
   - Female
   - Intersex
   - Other identity, specify:_________

2. What is your birth date/birthday?

   Month Day Year

3. How old were you at your last birthday?

   years old

4. Do you consider yourself
   - Heterosexual/Straight
   - Homosexual – Gay male
   - Homosexual – Lesbian
   - Bisexual
   - Other specify) _____________

5. Are you **currently** married or living with man/woman with whom you currently have a sexual relationship?
   - Married, living with spouse
   - Married, living with other sexual partner
   - Married, not living with spouse or other sexual partner
   - Not married, living with sexual partner
   - Not married, not living with sexual partner

6. What is the highest level of school you completed?
   - None
   - Primary (4 classes)
   - Secondary (8 classes)
   - Higher (12 classes)
   - University
   - Post-University

7. How many total years of education have you completed?

8. What religion are you?
   - Muslim
   - Catholic
   - Orthodox
   - No religion
   - Other:___________________

9. Where do you currently live?
   - In an apartment/house I own
   - In an apartment/house I rent
   - At my parent's/relative's apartment/house
   - Living/staying with someone (**not** a relative) & **not** paying rent
   - Homeless (on the street/in car)
   - Jail or correctional facility
   - Hospital/Institution
   - Other (specify)_________________

10. How long have you lived in Albania?

    Number of years

11. In the last 12 months have you been away from your home for more than one month altogether?
    - Yes
    - No
    - Don't know

12. What best describes your current job (work) situation?
    - Employed full-time (33-40 hours a week)
    - Employed part-time (less than 33 hours a week)
    - Not working – looking for work
    - Not working – student/homemaker/ other
    - Not working – not looking for work
    - Retired
    - Other(specify)____________________

13.
A) Have you ever been arrested and put in jail, detention or prison for longer than 24 hours?
   - Yes (**Go to question 13B**)
   - No (**Go to question 14**)

B) How many times have you been in jail, detention or prison for longer than 24 hours?

   ________times in jail

C) How old were you the first time this happened?

   ________ years old

CONTINUE ON TO THE NEXT PAGE

14. How often have you had a drink containing alcohol – a glass of beer, wine, a mixed drink, or any kind of alcoholic beverage – in the past 30 days? (**Check one**)
- ☐ Daily
- ☐ Nearly every day
- ☐ 3 or 4 times a week
- ☐ Once or twice a week
- ☐ 2 or 3 times a month
- ☐ Once a month
- ☐ Never

15. On days when you drank any alcoholic beverages, in the last 30 days, how many drinks did you usually have altogether? By a drink, we mean a can or glass of beer, a 4-ounce glass of wine, a 1½-ounce shot of liquor, or a mixed drink with 1½ ounces of liquor. (**Check one**)
- ☐ 1 – 2 drinks per day
- ☐ 3 – 4 drinks per day
- ☐ 5 – 6 drinks per day
- ☐ 7 – 8 drinks per day
- ☐ 9 – 11 drinks per day
- ☐ More than 12 drinks per day

16. During the past 30 days, how often have you had 5 or more drinks of alcohol in a row, that is within a couple of hours (e.g. 2-4 hours)?
- ☐ Daily
- ☐ Nearly every day
- ☐ 3 or 4 times a week
- ☐ Once or twice a week
- ☐ 2 or 3 times a month
- ☐ Once a month
- ☐ Never

17.
A) Have you **ever**-tried drugs that were not prescribed by a physician (for example – marijuana, cocaine, heroine, morphine, Valium)?
- ☐ Yes (**Go to question 17B**)
- ☐ No (**Go to question 18**)

B) If yes, have you used any of the drugs in the past 12 months?
- ☐ Yes
- ☐ No

C) Have you **ever** injected any of the drugs that you have tried?
- ☐ Yes
- ☐ No

D) Have you injected any drugs in the past 12 months?
- ☐ Yes
- ☐ No

18. What was the month and year that you first tested positive for HIV?

_____ ______
month year

19. After receiving your HIV diagnosis, when did you have your first visit with a doctor for your HIV/AIDS?
- ☐ Within one month after diagnosis
- ☐ One to three months after diagnosis
- ☐ Four to six months after diagnosis
- ☐ Seven months to a year after diagnosis
- ☐ More than a year after diagnosis

20. What is (are) the most likely way(s) that you became infected with HIV? (**Select all that apply**)
- ☐ Sex with a man who was HIV+
- ☐ Sex with a woman who was HIV+
- ☐ Shared needles with a person who was HIV+
- ☐ Blood transfusion or other medical procedure
- ☐ Acquired at birth
- ☐ Don't know
- ☐ Other (needle stick at work, etc.)
  Specify___________________

21. Have you been diagnosed with any of the following diseases listed below? (**Select all that apply**)

	Yes	No	Don't know
Pneumonia	☐	☐	☐
Tuberculosis	☐	☐	☐
Hepatitis A or B	☐	☐	☐
Hepatitis C	☐	☐	☐
Syphilis	☐	☐	☐
Herpes (genital)	☐	☐	☐
Gonorrhea	☐	☐	☐
Chlamydia	☐	☐	☐
Genital warts	☐	☐	☐
Yeast infections	☐	☐	☐
Other (specify)___________________			

22. In general, would you say that today your physical health is:
- ☐ Excellent
- ☐ Good
- ☐ Fair
- ☐ Good

CONTINUE ON TO THE NEXT PAGE

23. How would you rate your <u>physical</u> health now as compared to when you first sought treatment for your HIV infection?

- ☐ Much better
- ☐ A little better
- ☐ About the same
- ☐ A little worse
- ☐ Much worse

24. In general, would you say that today your <u>emotional</u> health is:

- ☐ Excellent
- ☐ Good
- ☐ Fair
- ☐ Poor

25. How would you rate your <u>emotional</u> health now as compared to when you first sought treatment for your HIV infection?

- ☐ Much better
- ☐ A little better
- ☐ About the same
- ☐ A little worse
- ☐ Much worse

26. At any time since diagnosis of your HIV infection have you been diagnosed with any of the following? (**Select all that apply**)

	Yes	No
Anxiety	☐	☐
Bipolar Disorder	☐	☐
Dementia	☐	☐
Depression	☐	☐
Other (specify)_______________		

27.

A) Is anyone else in your household that is HIV positive?

- ☐ Yes (**Go to question 27B**)
- ☐ No (**End**)

B) If yes, are they currently on medication for HIV?

- ☐ Yes
- ☐ No

THANK YOU

END
